# Supplementary material for: Inhibition of the CDK2 and Cyclin A complex leads to autophagic degradation of CDK2 in cancer cells
Source: Nat Commun. 2022 May 20;13:2835. doi: 10.1038/s41467-022-30264-0 (PMC9122913; doi:10.1038/s41467-022-30264-0)
Supplement: Supplementary file 1 — Supplementary Information [file 41467_2022_30264_MOESM1_ESM.pdf]

Table S1. CDK2 druggability prediction.

No.	Pred. Max pKd	Pred. Avg pKd	DrugScore	Druggability	Residues
1	10.45	6.93	970	Druggable	Cavity1|HIS:119:A ARG:122:A ALA:151:A PHE:152:A GLY:153:A VAL:154:A PRO:155:A VAL:156:A ARG:157:A THR:158:A GLU:172:A ILE:173:A GLY:176:A CYS:177:A LYS:178:A TYR:179:A TYR:180:A SER:181:A THR:182:A ALA:183:A VAL:184:A TRP:227:A PRO:228:A MET:233:A TYR:269:A ASP:270:A PRO:271:A ASN:272:A LYS:273:A ARG:274:A ASN:173:B GLU:174:B VAL:175:B PRO:176:B ASP:177:B TYR:178:B HIS:179:B GLU:180:B ILE:182:B HIS:183:B LEU:186:B GLN:228:B ASN:229:B GLU:230:B THR:231:B GLU:268:B ILE:270:B ASN:312:B GLN:313:B LEU:315:B THR:316:B GLN:317:B TYR:318:B PHE:319:B LEU:320:B HIS:321:B GLN:322:B GLN:323:B PRO:324:B ALA:325:B ASN:326:B CYS:327:B LYS:328:B VAL:329:B GLU:330:B SER:331:B LEU:332:B MET:334:B LYS:379:B TYR:413:B SER:416:B LYS:417:B TYR:418:B HIS:419:B
2	11.2	6.98	1255	Druggable	Cavity2|ARG:50:A LEU:54:A HIS:119:A ARG:122:A ARG:126:A ALA:149:A ARG:150:A ALA:151:A PHE:152:A GLY:153:A VAL:154:A PRO:155:A VAL:156:A ARG:157:A THR:158:A TYR:159:A THR:160:A CYS:177:A LYS:178:A TYR:179:A TYR:180:A SER:181:A THR:182:A TRP:227:A ASP:270:A PRO:271:A ASN:272:A ASN:173:B GLU:174:B VAL:175:B PRO:176:B ASP:177:B TYR:178:B HIS:179:B GLU:180:B ILE:182:B HIS:183:B LEU:186:B VAL:219:B GLY:222:B GLU:223:B GLU:224:B TYR:225:B LYS:226:B LEU:227:B GLN:228:B ASN:229:B GLU:230:B THR:231:B LEU:234:B SER:265:B LYS:266:B PHE:267:B GLU:268:B GLU:269:B ILE:270:B TYR:271:B PRO:272:B PRO:273:B ALA:307:B ASN:312:B GLN:313:B LEU:315:B THR:316:B GLN:317:B TYR:318:B PHE:319:B LEU:320:B HIS:321:B GLN:322:B GLN:323:B PRO:324:B ALA:325:B ASN:326:B CYS:327:B LYS:328:B VAL:329:B GLU:330:B SER:331:B LEU:332:B MET:334:B LYS:412:B TYR:413:B ASN:415:B SER:416:B LYS:417:B TYR:418:B HIS:419:B
3	11.52	6.93	756	Druggable	Cavity3|GLU:8:A LYS:9:A ILE:10:A GLY:11:A GLU:12:A GLY:13:A THR:14:A TYR:15:A GLY:16:A VAL:17:A VAL:18:A TYR:19:A LYS:20:A VAL:30:A ALA:31:A LEU:32:A LYS:33:A LYS:34:A ILE:35:A THR:47:A GLU:51:A LEU:55:A VAL:64:A LYS:65:A LEU:66:A LEU:78:A PHE:80:A GLU:81:A PHE:82:A LEU:83:A HIS:84:A GLN:85:A ASP:86:A LEU:87:A LYS:88:A LYS:89:A PHE:90:A ASP:127:A LYS:129:A PRO:130:A GLN:131:A ASN:132:A LEU:133:A LEU:134:A ILE:135:A LEU:143:A ALA:144:A ASP:145:A PHE:146:A GLY:147:A LEU:148:A ALA:149:A VAL:163:A LEU:296:A LEU:298:A
4	11.31	6.49	291	less druggable	Cavity4|LEU:186:B MET:189:B GLU:190:B VAL:191:B LYS:192:B CYS:193:B LYS:194:B PRO:195:B ARG:211:B LEU:232:B HIS:233:B LEU:234:B ALA:235:B VAL:236:B ASN:237:B TYR:238:B ILE:239:B ASP:240:B ARG:241:B ALA:308:B PRO:309:B THR:310:B VAL:311:B ASN:312:B GLY:337:B GLU:338:B LEU:339:B SER:340:B LEU:341:B ILE:342:B ASP:343:B ALA:344:B ASP:345:B PRO:346:B TYR:347:B LEU:348:B LYS:349:B TYR:350:B LEU:351:B PRO:352:B SER:353:B ILE:355:B
5	9.9	6.01	-598	Undruggable	Cavity5|ILE:206:B THR:207:B SER:209:B MET:210:B ARG:211:B ALA:212:B ILE:213:B LEU:214:B VAL:215:B ASP:216:B TRP:217:B LEU:218:B GLU:220:B LEU:249:B ARG:250:B GLY:251:B LYS:252:B LEU:253:B GLN:254:B LEU:255:B ILE:281:B THR:282:B ASP:283:B THR:285:B TYR:286:B
6	9.02	5.71	-807	Undruggable	Cavity6|MET:1:A LEU:37:A ILE:52:A SER:53:A LEU:55:A LYS:56:A GLU:57:A ASN:59:A LYS:65:A LEU:66:A LEU:67:A ASP:68:A VAL:69:A ILE:70:A HIS:71:A THR:72:A GLU:73:A LEU:76:A VAL:79:A ARG:293:B GLU:295:B HIS:296:B LEU:297:B VAL:298:B LEU:299:B LYS:300:B VAL:301:B LEU:302:B THR:303:B PHE:304:B ASP:305:B
7	8.44	5.51	-655	Undruggable	Cavity7|TRP:167:A ALA:194:A GLU:195:A VAL:197:A THR:198:A ARG:199:A ARG:200:A ALA:201:A LEU:202:A PHE:203:A PRO:204:A GLY:205:A PHE:213:A ARG:214:A ILE:215:A PHE:216:A ARG:217:A THR:218:A LEU:219:A GLN:246:A ASP:247:A SER:249:A LYS:250:A VAL:251:A VAL:252:A PRO:253:A PRO:254:A
8	8.26	5.45	-807	Undruggable	Cavity8|ARG:126:A ARG:157:A THR:158:A TYR:159:A THR:160:A HIS:161:A GLU:162:A VAL:163:A VAL:164:A LEU:166:A ARG:169:A ALA:170:A GLU:172:A ILE:173:A LEU:174:A LEU:175:A GLY:176:A CYS:177:A LYS:178:A TYR:179:A TYR:180:A GLU:208:A ILE:209:A LEU:212:A PRO:234:A ASP:235:A
9	8.13	5.41	-353	Undruggable	Cavity9|LEU:87:A LYS:88:A LYS:89:A PHE:90:A MET:91:A ASP:92:A ALA:93:A SER:94:A ALA:95:A LEU:96:A THR:97:A GLY:98:A ILE:99:A ILE:104:A LYS:129:A PRO:130:A GLN:131:A ASN:132:A THR:165:A LEU:166:A TRP:167:A TYR:168:A ILE:192:A GLU:195:A MET:196:A VAL:197:A THR:198:A ARG:199:A ARG:200:A ALA:201:A LEU:202:A PRO:204:A
10	7.73	5.27	-1264	Undruggable	Cavity10|LYS:20:A GLY:27:A GLU:28:A VAL:29:A VAL:30:A PRO:61:A LYS:65:A LEU:67:A PHE:80:A GLU:81:A PHE:82:A LEU:83:A HIS:84:A ILE:135:A ASN:136:A THR:137:A GLU:138:A GLY:139:A ALA:140:A LYS:142:A

Table S2. Top 10 drug molecules with potential high affinity to cyclin-CDK2 interface.

Rank	Accession Number	Drug Name	Structure
1	DB03147	Flavin adenine dinucleotide	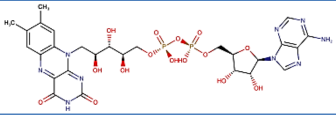
2	DB00410	Mupirocin	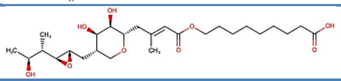
3	DB06441	Cangrelor	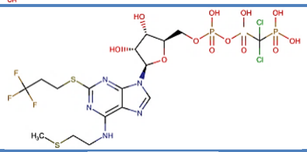
4	DB04865	Homoharringtonine	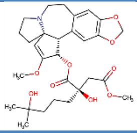
5	DB00819	Acetazolamide	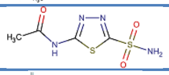
6	DB09341	Dextrose	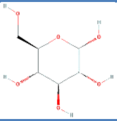
7	DB00703	Methazolamide	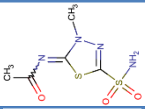
8	DB01077	Etidronic acid	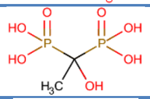
9	DB00147	Pyridoxal	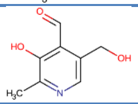
10	DB04398	Lactic Acid	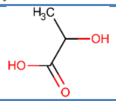

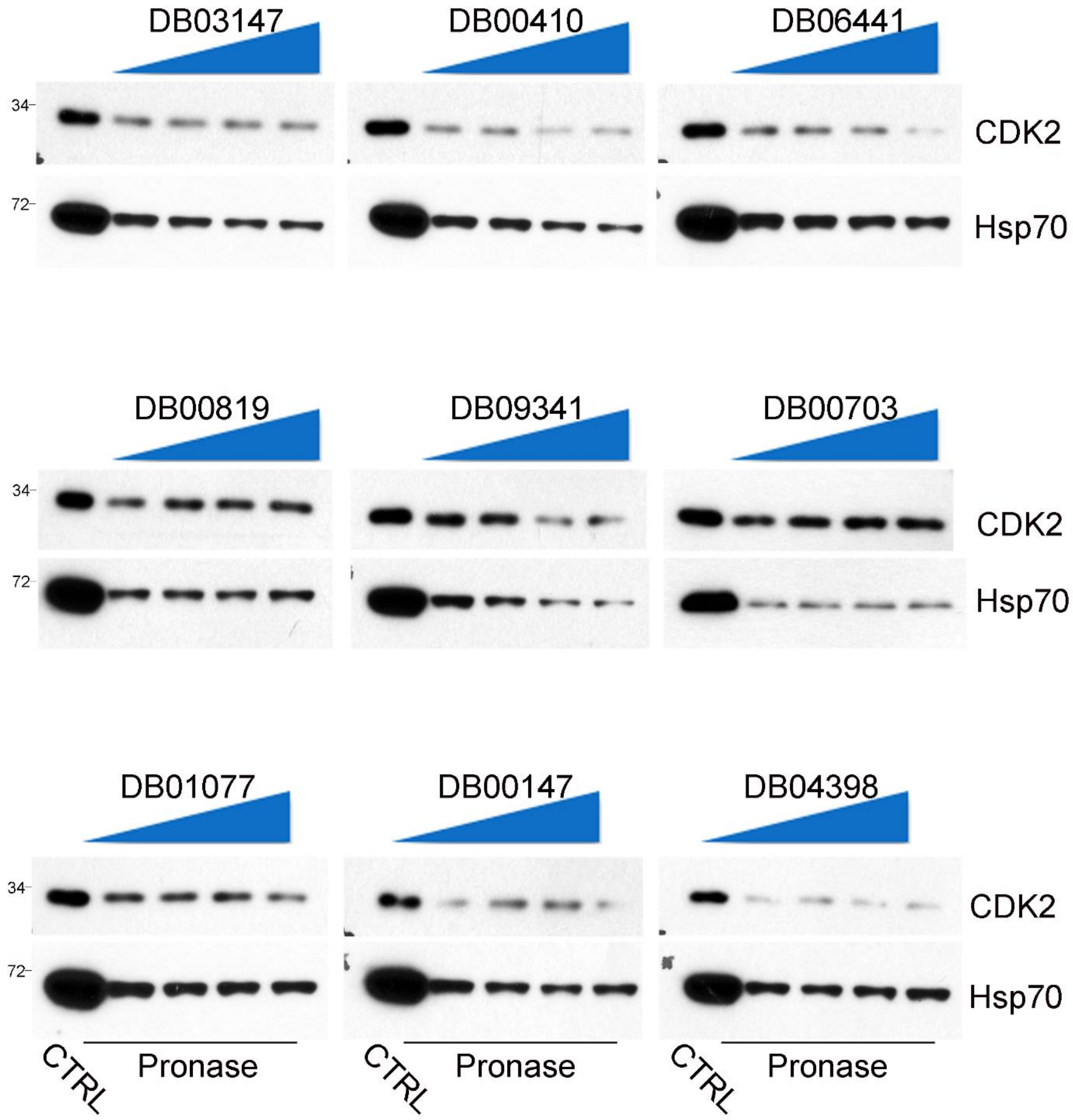

Fig. S1. DARTS assay to identify the interaction between CDK2 and compounds listed in SI Table 1. CTRL: no pronase treatment. Blue triangle represents 10 times increased dose of indicated compound (0 μM, 1 μM, 10 μM, 100 μM). All the western-blotting results shown here were representative of three independent experiments. Source data are provided as a Source Data file.

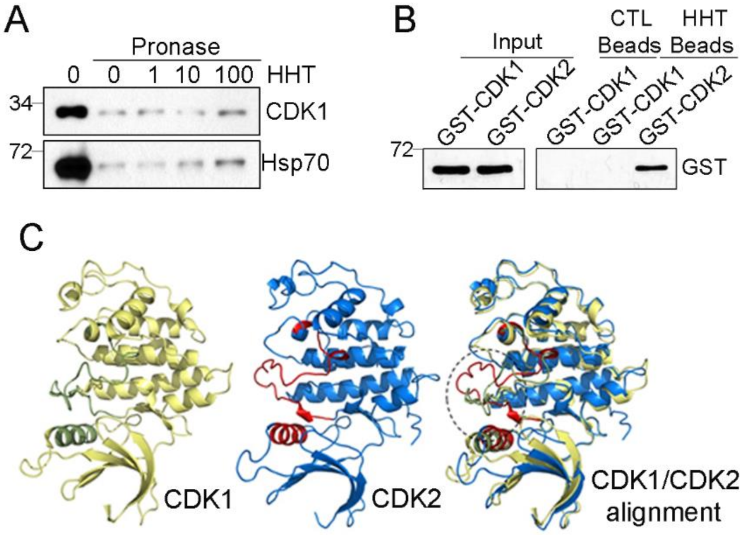

Fig. S2. The interaction between CDK1 and HHT. (A) DARTS assay to identify the interaction between HHT (ng/mL) and CDK1. (B) Pulldown assay by HHT conjugated magnetic beads with purified GST-CDK1 or GST-CDK2 proteins. The western-blotting results shown here were representative of three independent experiments. (C) The structure alignment between CDK1 and CDK2 protein. The cartoon shows the subunit structure of CDK1 (yellow orange), CDK2 (blue) and alignment. The C-helix and activation segment of CDK1 and CDK2 are colored in smudge and red, respectively. The dark circle area demonstrates the different structure between CDK1 and CDK2. Source data are provided as a Source Data file.

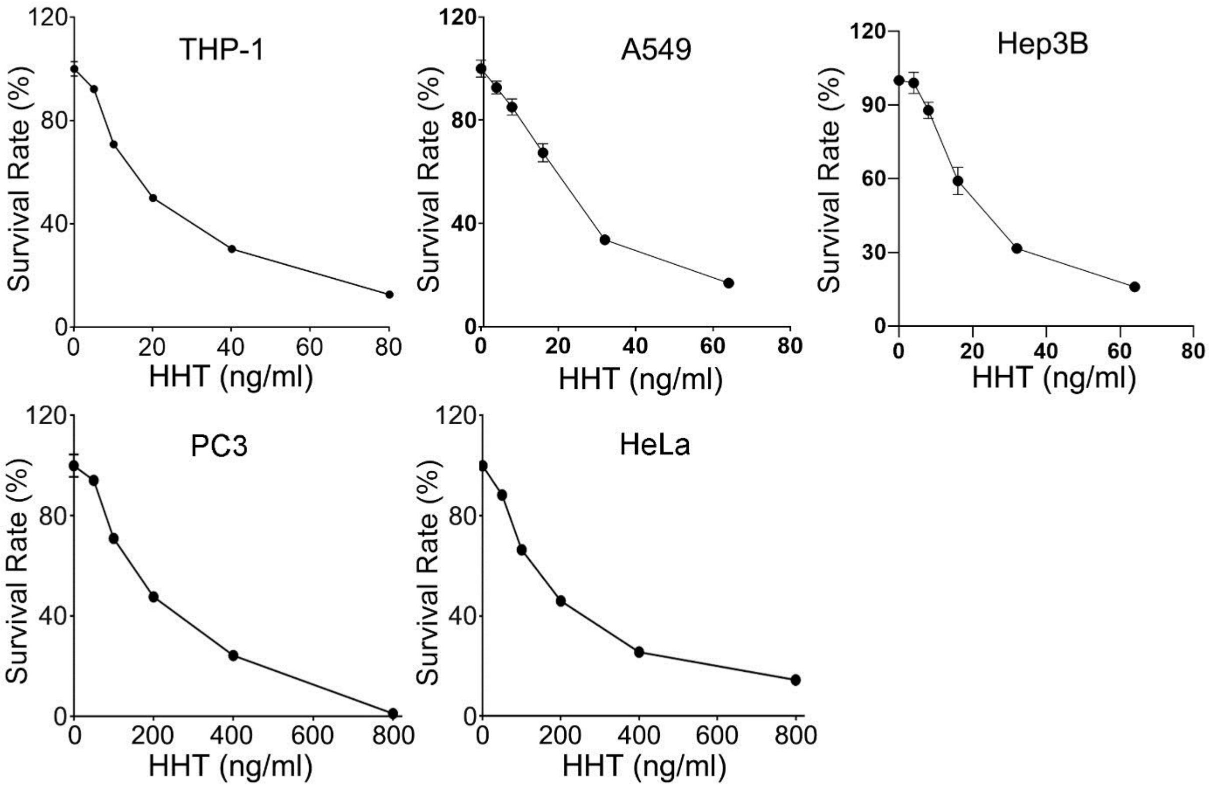

Fig. S3. Cell survival curve from MTS assay for different cell lines treated with HHT for 24 hours. The results shown here were representative of three independent experiments. Data are expressed as mean + /- s.d. of independent experiments (n = 4 for THP-1, PC3 and HeLa; n=6 for A549 and Hep3B). Source data are provided as a Source Data file.

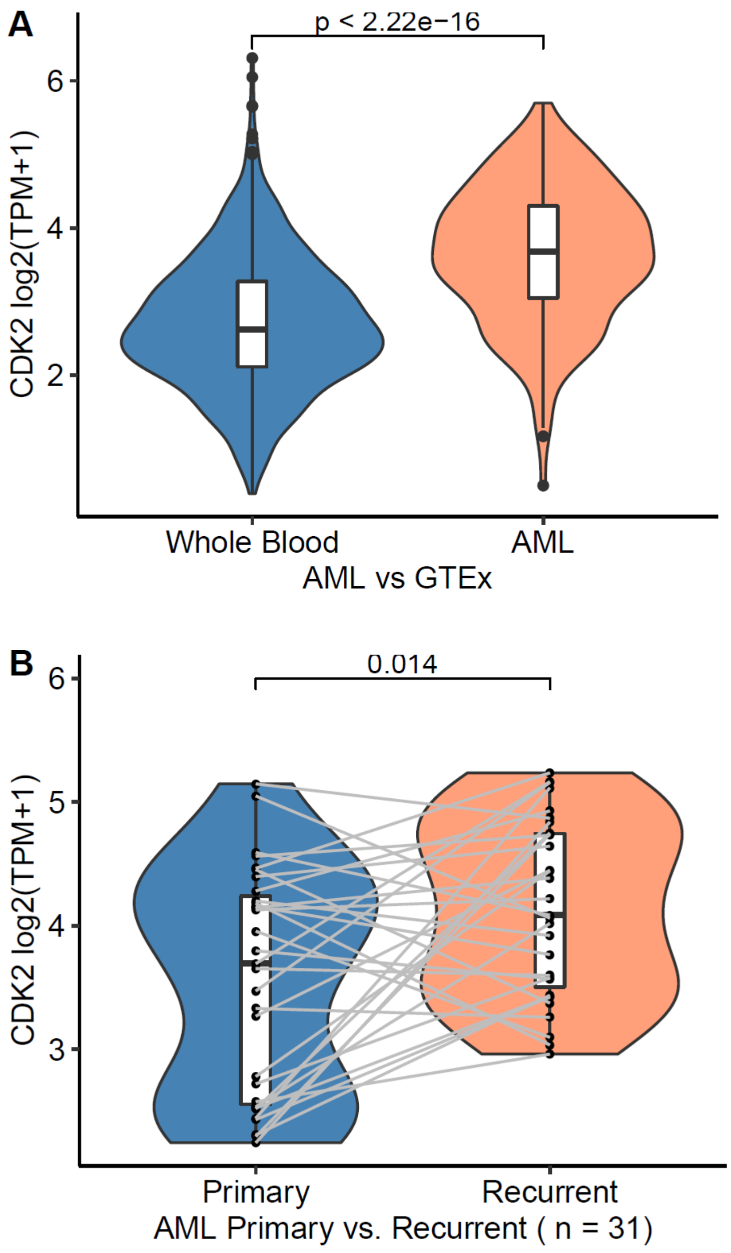

Fig. S4. High expression level of CDK2 were significant enriched in AML cohort. (A) Expression level of *CDK2* in TARGET-AML (n=156) and GTEx cohort (n=1582). (B) Expression of CDK2 in cases with both primary and recurred samples using TARGET-AML (n = 31). Box boundaries in (A) and (B) are the 25th and 75th percentiles, the horizontal line across the box is the median, and the whiskers indicate the minimum and maximum values. Wilcoxon Test were used to statistical analysis for (A) and (B).

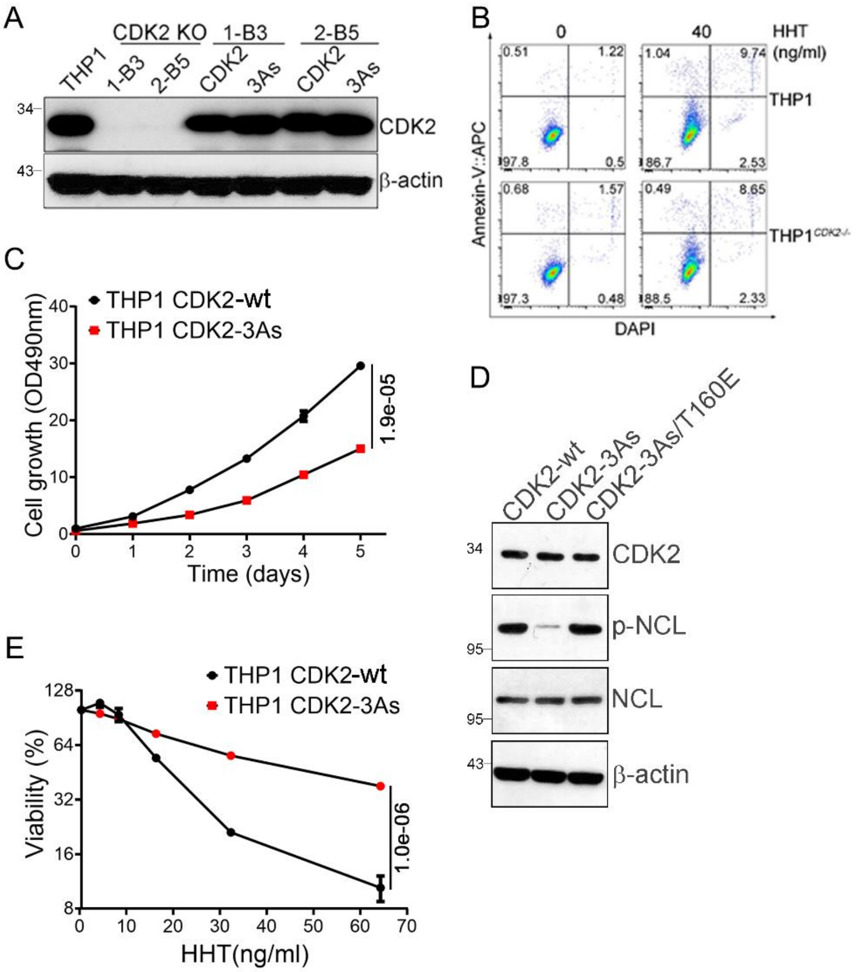

Fig. S5. The effects of CDK2 mutants on THP1 cell prolifeation and response to HHT treatment. (A) Representative western blots of CDK2 protein in THP1 CDK2 knockout cells and THP1 CDK2-KO cells with re-expression of CDK2 or CDK2-3As. (B) Representative result of Annexin-V/DAPI staining assay to detect the apoptotic rate in THP1 and THP1CDK2-/- cells with HHT treatment for 24 hours and the negative control with DMSO treatment. (C) Growth curves of THP1 CDK2 and THP1 CDK2-3As cells. (D) Representative western blots of CDK2 downstream target protein-NCL in THP1 CDK2-wt, CDK2-3As and CDK2-3As/T160E cells. (E) Cell survival curves of THP1 CDK2 and THP1 CDK2-3As cells with HHT treatment. Data represent the mean ± SD from four independent biological samples for each group for (C and E). P values are indicated by two-tailed unpaired Student's t test for (C and E).

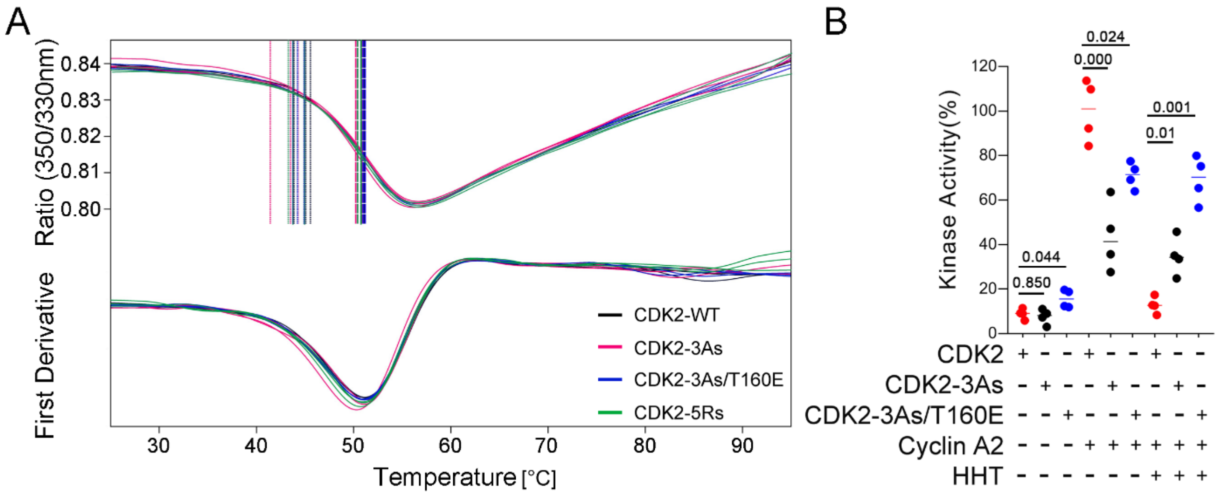

Fig S6. NanoDSF and Kinase activity analysis of CDK2 and its mutants. (A) nanoDSF traces with the relative first derivatives for CDK2 and the mutants. X-axis represents temperature in ℃ and y-axis represents first derivative of ratio of intrinsic fluorescence (350:330 nm) .The black, red, blue and green solid lines show the curves of wild-CDK2, CDK2-3As, CDK2-3As/T160E and CDK2-5Rs measured in assay buffer (150 mM NaCl, 100 mM Tris, pH 7.5). Three replicates for each sample. (B) The kinase activity assay for wild-CDK2, CDK2-3As and CDK2-3As/T160E protein with the presence of cyclin A2 (20 ng) and HHT (100 ng/mL) or not. Data represent the mean ± SD from four independent biological samples for each group. P values are indicated by one-way ANOVA with Tukey's multiple comparison test. Source data are provided as a Source Data file.

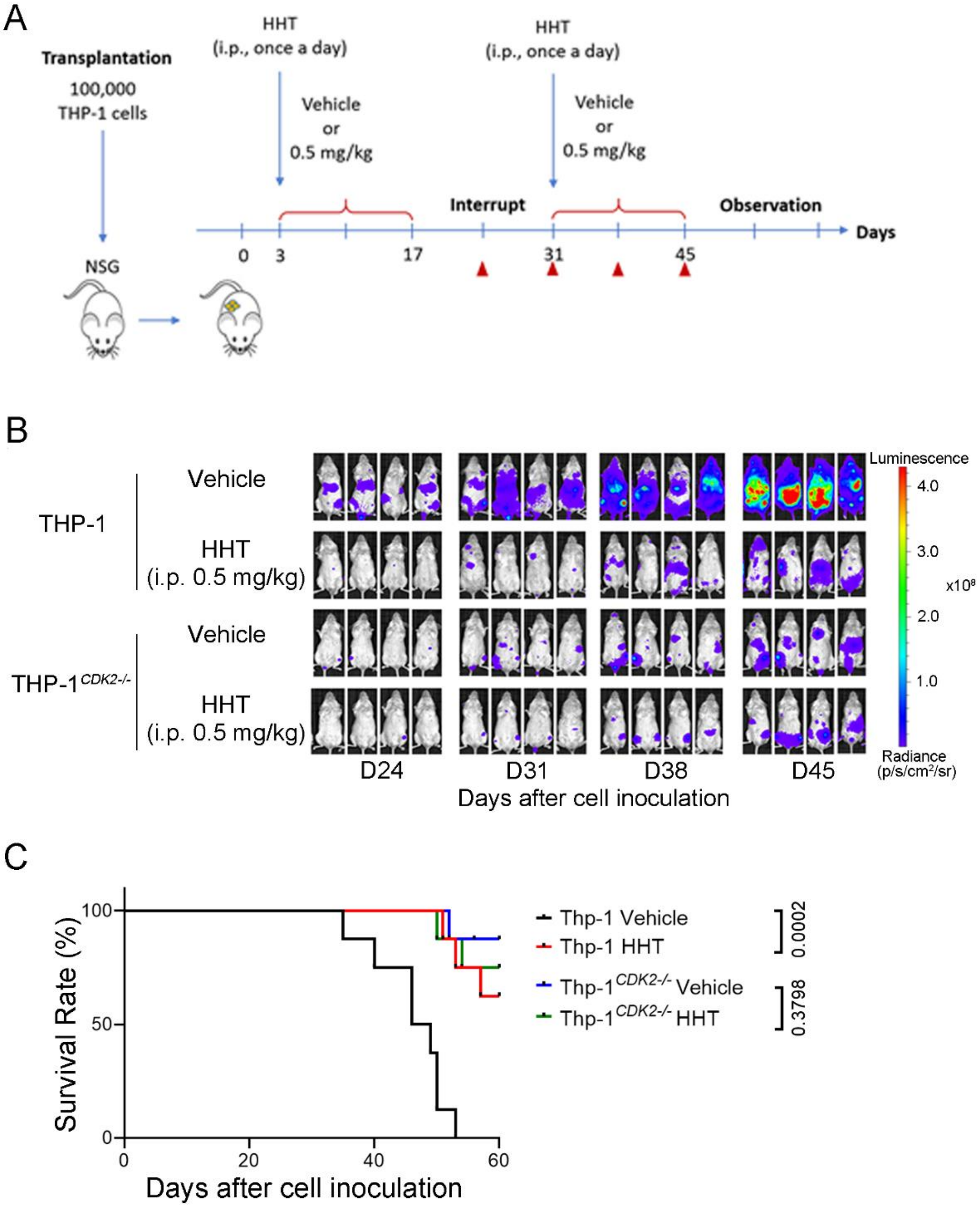

Fig. S7. Leukemia cells xenograft model. (A) Scheme of the THP1 xenograft model and pharmacological inhibition by HHT. (B) Bioluminescence imaging of luciferase-expressing THP1 (CDK2 wild-type or knockout) xenograft mice models, treated with 0.5 mg/kg HHT (n=6). (C) Kaplan–Meier survival of luciferase-expressing THP1(CDK2 wild-type or knockout) xenograft mice models, treated with 0.5 mg/kg HHT or vehicle as the control (n = 10 mice per group). The p value was calculated by log-rank test.

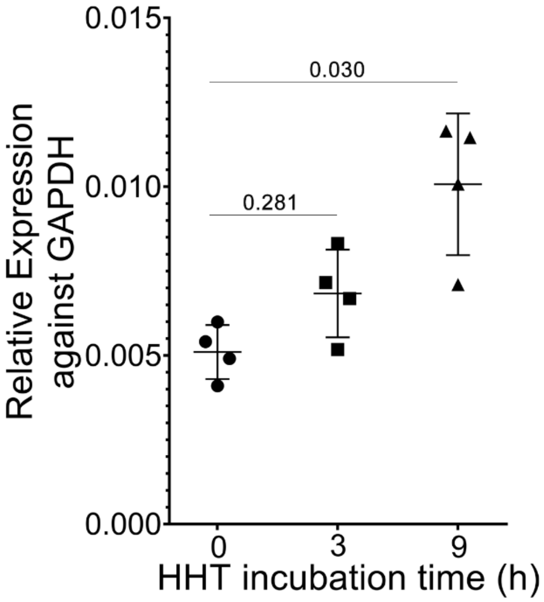

Fig. S8. CDK2 mRNA level in THP1 cells after 40 ng/mL of HHT treatment. Data are expressed as mean + /- s.d. of independent experiments (n = 4). Data represent the mean ± SD from four independent biological samples for each group. P values are indicated by one-way ANOVA with Tukey's multiple comparison test. Source data are provided as a Source Data file.

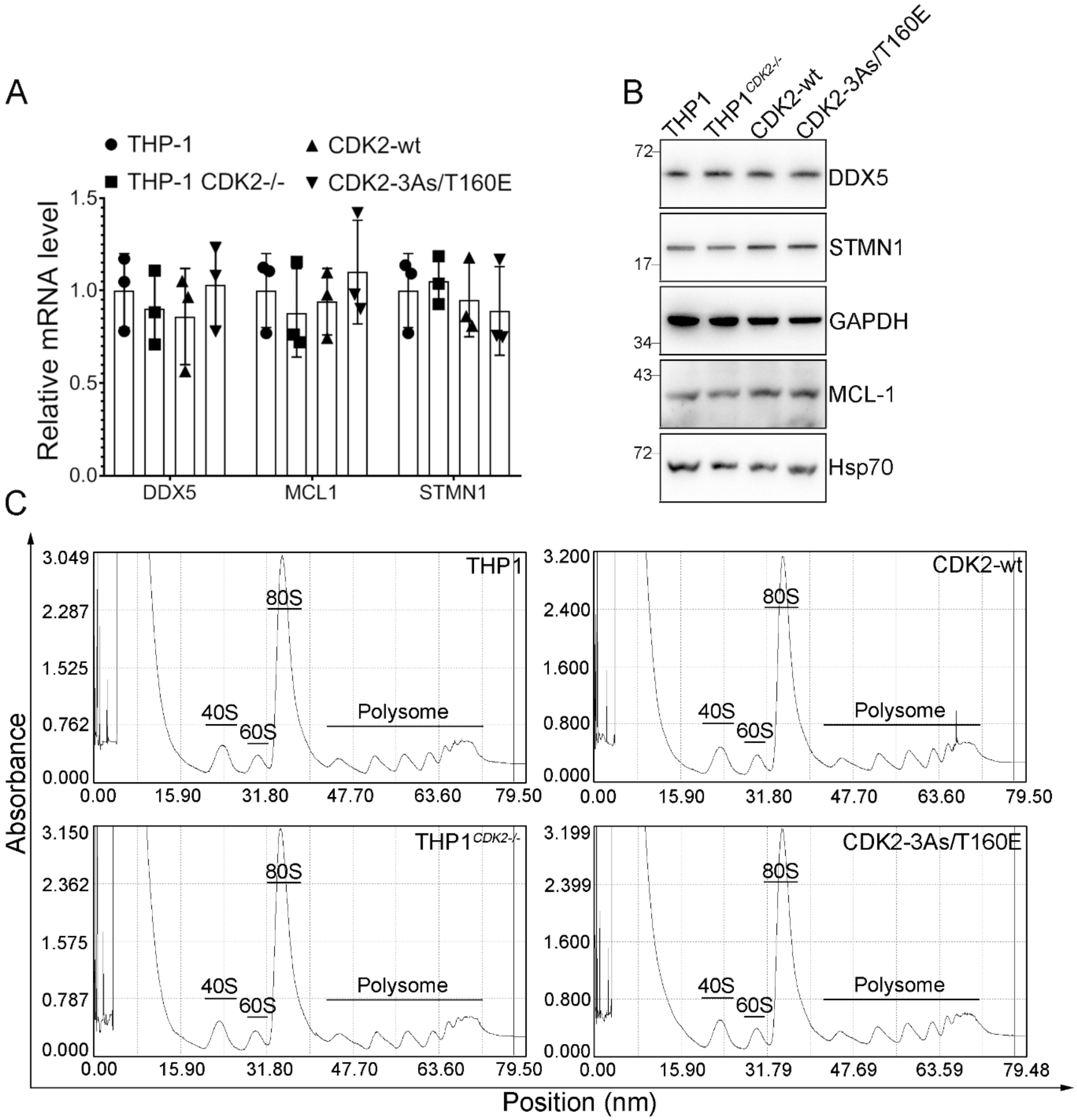

Fig. S9. The effect of CDK2 on mRNA translation. The mRNA (A) and protein (B) level of DDX5, MCL1 and STMN1 in THP1 cells with different CDK2 status. Data represent the mean + SD from four independent biological samples for each group for (A). P values are indicated by one-way ANOVA with Tukey's multiple comparison test for (A), and there is no statistical significance among these groups for each gene expression level. Source data are provided as a Source Data file. (C)The ribosome profiling of THP1 cells with different CDK2 status. The results shown here were representative of three independent experiments. Source data are provided as a Source Data file.

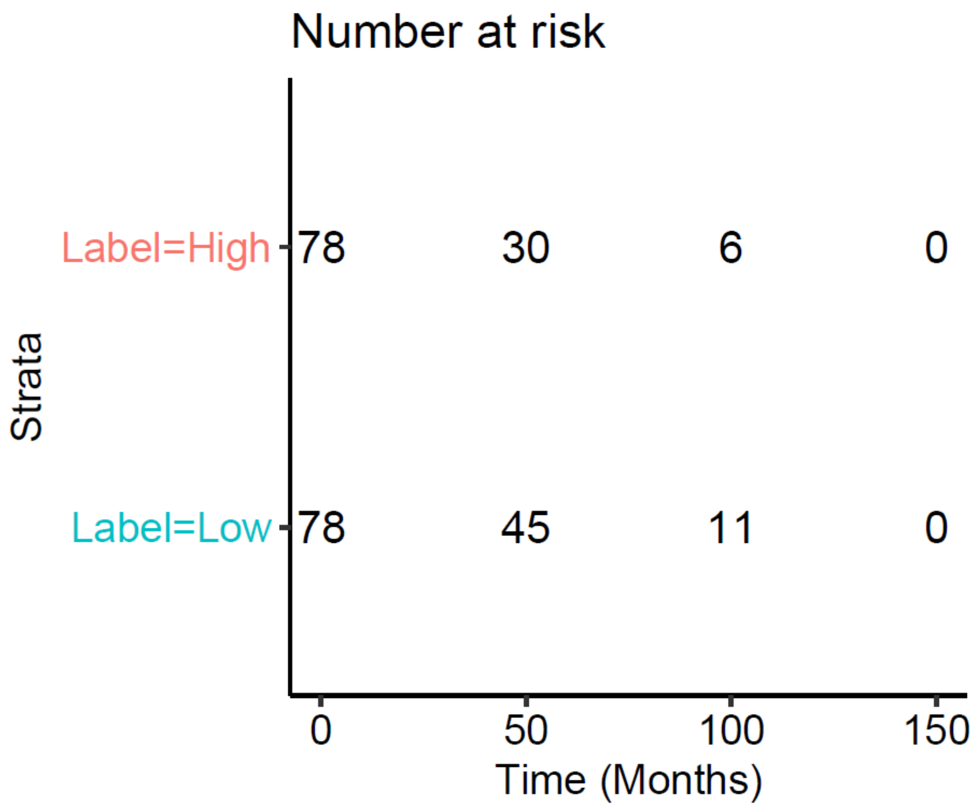

Fig. S10. The detailed risk table in survival analysis. The patients were dichotomized on the basis of the median value of CDK2 mRNA expression. Wilcoxon Test was used to estimate difference in expression level, and Log Rank test was used to survival analysis.

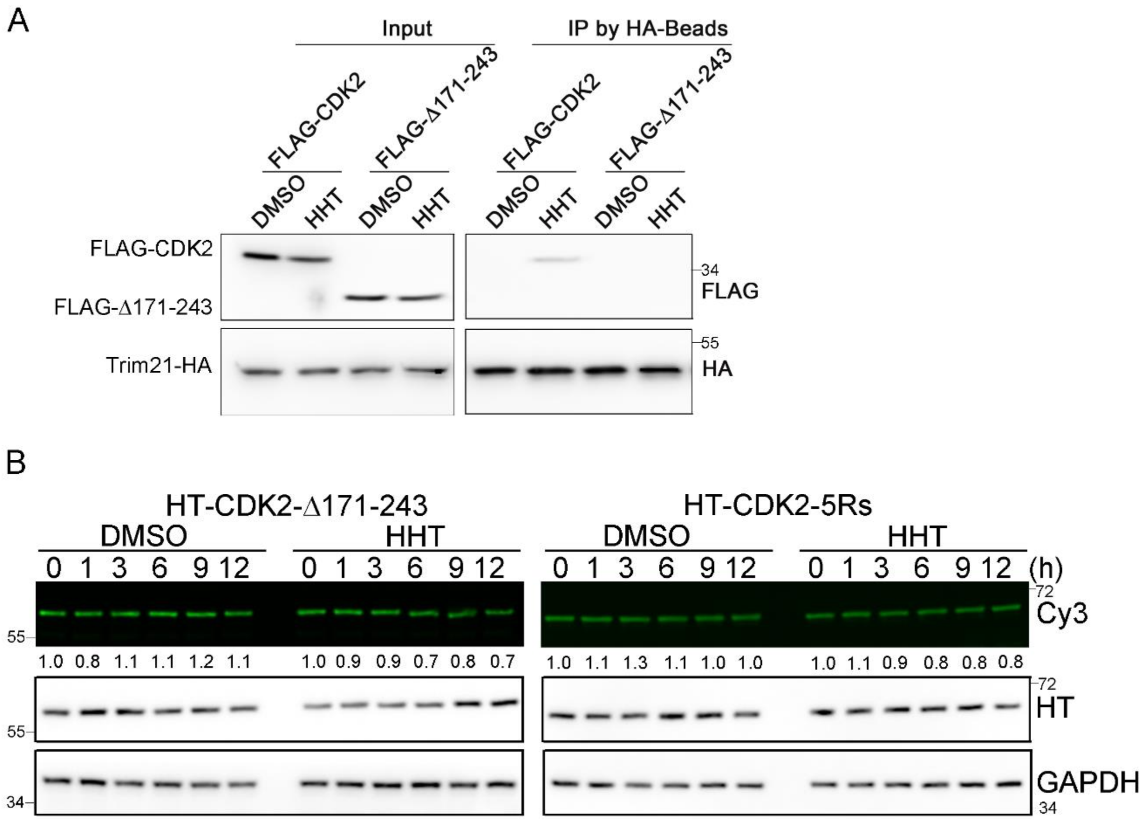

Fig. S11. The interaction of CDK2 deletion mutant with Trim21 and the response of different CDK2 mutants to HHT treatment. (A) HEK293 cells transfected with Trim21-HA plasmid and 3xFLAG-CDK2 or 3xFLAG-CDK2-Δ171-243 plasmid were treated with 100 ng/mL of HHT for 6 hours, followed by co-immunoprecipitation with HA-beads; protein levels of 3xFLAG-CDK2, 3xFLAG-CDK2-Δ171-243 and Trim21-HA were analyzed by western blot. (B) Pulse-chase analysis of different CDK2 mutants using HT-TMR system in 293T cells transfected with HT-CDK2-Δ171-243 (left panel) or HT-CDK2-5Rs (right panel). The HT-TMR ligand-labeled HT-CDK2 was visualized with a fluoro-image analyzer after the treatment with HHT at 100 ng/mL. The total level of HT-CDK2-5Rs and HT-CDK2-Δ171-243 were determined by western blot with GAPDH as the loading control. The western-blotting results shown here were representative of three independent experiments.

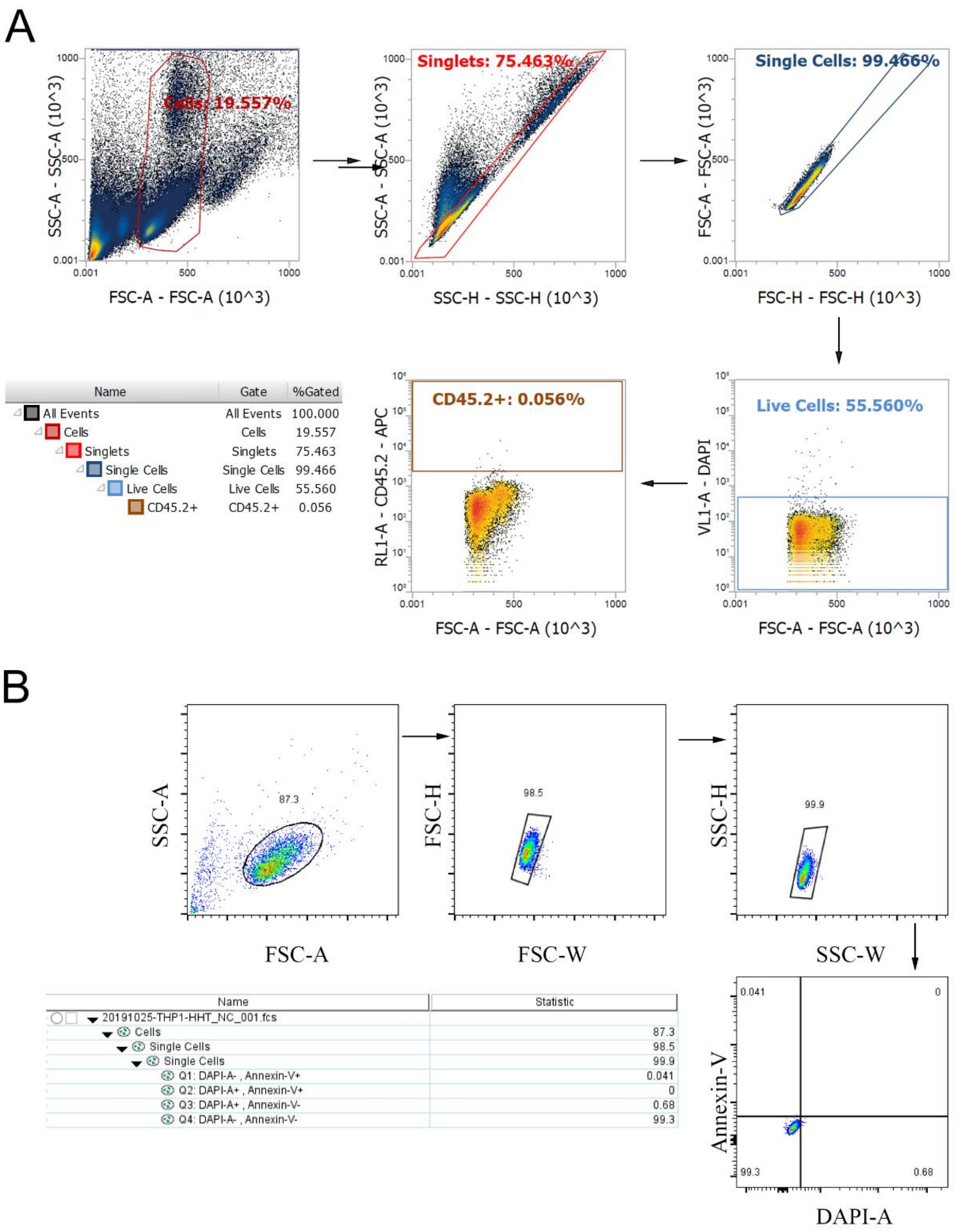

Fig. S12. The FACS gating panels for Fig 4B (A) and Fig S5B (B).
