# Supplementary material for: Inhibition of the CDK2 and Cyclin A complex leads to autophagic degradation of CDK2 in cancer cells
Source: Nat Commun. 2022 May 20;13:2835. doi: 10.1038/s41467-022-30264-0 (PMC9122913; doi:10.1038/s41467-022-30264-0)
Supplement: Supplementary file 4 — Reporting Summary [file 41467_2022_30264_MOESM4_ESM.pdf]

nature portfolio

Corresponding author(s): Wendong Hung

Last updated by author(s): Mar 31, 2022

Reporting Summary

Nature Portfolio wishes to improve the reproducibility of the work that we publish. This form provides structure for consistency and transparency in reporting. For further information on Nature Portfolio policies, see our Editorial Policies and the Editorial Policy Checklist.

Statistics

For all statistical analyses, confirm that the following items are present in the figure legend, table legend, main text, or Methods section.

n/a	Confirmed	
☐	☒	The exact sample size (*n*) for each experimental group/condition, given as a discrete number and unit of measurement
☐	☒	A statement on whether measurements were taken from distinct samples or whether the same sample was measured repeatedly
☐	☒	The statistical test(s) used AND whether they are one- or two-sided<br>*Only common tests should be described solely by name; describe more complex techniques in the Methods section.*
☐	☒	A description of all covariates tested
☐	☒	A description of any assumptions or corrections, such as tests of normality and adjustment for multiple comparisons
☐	☒	A full description of the statistical parameters including central tendency (e.g. means) or other basic estimates (e.g. regression coefficient) AND variation (e.g. standard deviation) or associated estimates of uncertainty (e.g. confidence intervals)
☐	☒	For null hypothesis testing, the test statistic (e.g. *F*, *t*, *r*) with confidence intervals, effect sizes, degrees of freedom and *P* value noted<br>*Give P values as exact values whenever suitable.*
☒	☐	For Bayesian analysis, information on the choice of priors and Markov chain Monte Carlo settings
☐	☒	For hierarchical and complex designs, identification of the appropriate level for tests and full reporting of outcomes
☒	☐	Estimates of effect sizes (e.g. Cohen's *d*, Pearson's *r*), indicating how they were calculated

Our web collection on statistics for biologists contains articles on many of the points above.

Software and code

Policy information about availability of computer code

Data collection	Data collection was done with BD CellQuest Pro software v.5.2 (BD Biosciences) for the flow cytometry experiment.<br>Data collection was done with BZ-X800 Viewer/Analyzer software v.1.1.1.8 (KEYENCE) for the Immunofluorescence experiment.<br>Data collection was done with Amersham Imager 680 v.2.0 (GE) for the Halo Tag experiment.<br>Data collection was done with Prometheus NT.48 instrument for the Nano Differential Scanning Fluorimetry experiment.<br>Data collection was done with Isco Density Gradient Fractionation System for the Ribosome profiling experiment.
Data analysis	ImageJ (version 2.0.0-rc-69/l.52n) for image analysis; Prism 7 (version 8.0.0) for statistical analysis; FlowJo (vl0.0.5) for flow cytometry I analysis; ThermControl v2.0.4 software (NanoTemper Techonlogies) for the Nano Differential Scanning Fluorimetry.<br>As a multiple-stage program for virtual ligand screening, the LIVS pipeline uses the three HTVS/SP/XP precisions of Glide software for docking in series. LIVS provides full-coverage and docks every compound in the library. To control the qualities of output compounds, this automatic program integrates Lipinski's rule of five, HTS frequent hitter (PAINS), protein reactive chemicals such as oxidizer or alkylator (ALARM), and maximization of the molecule diversity by using UDScore (Universe Diversity Score, developed by us to measure library diversity which is independent of library size).<br>Western blot images and micrograph images were assembled in Adobe Photoshop 2020 version 21.1.2.<br>qPCR data was analyzed in Microsoft Excel version 16.36.<br>the protein-protein interaction model of CDK2 (3-dimensional structure is extracted from PDB code: 1FIN) and C-terminal region of Trim21 (PDB -code: 2IWG) were predicted using Zdock software v.3.0.1.<br>The binding mode of HHT was calculated by our in-house developed AAD (All-Around Docking) program.<br>Custom code is available upon reasonable request.

For manuscripts utilizing custom algorithms or software that are central to the research but not yet described in published literature, software must be made available to editors and reviewers. We strongly encourage code deposition in a community repository (e.g. GitHub). See the Nature Portfolio guidelines for submitting code & software for further information.

Data

Policy information about availability of data

All manuscripts must include a data availability statement. This statement should provide the following information, where applicable:

- Accession codes, unique identifiers, or web links for publicly available datasets
- A description of any restrictions on data availability
- For clinical datasets or third party data, please ensure that the statement adheres to our policy

The authors declare that all data supporting the findings of this study are available within the paper and its Supplementary Information files.
The sgRNA sequences targeting human CDK2 were designed using the CRISPR on-line design tool (www.genome-engineering.org/crispr).
The mRNA expression and survival results presented in this study are based upon data generated by the Therapeutically Applicable Research to Generate Effective Treatments (https://ocg.cancer.gov/programs/target) initiative, phs000465. The data used for this analysis are available at https://portal.gdc.cancer.gov/projects.
The structure data used in this study are available in the PDB database under accession codes 1FIN (https://www.rcsb.org/structure/1FIN),2IWG (https://www.rcsb.org/structure/2IWG),4Y72(https://www.rcsb.org/structure/4Y72).

Field-specific reporting

Please select the one below that is the best fit for your research. If you are not sure, read the appropriate sections before making your selection.

☒ Life sciences ☐ Behavioural & social sciences ☐ Ecological, evolutionary & environmental sciences

For a reference copy of the document with all sections, see nature.com/documents/nr-reporting-summary-flat.pdf

Life sciences study design

All studies must disclose on these points even when the disclosure is negative.

Sample size	No statistical methods were used to predetermine sample size. Sample sizes were determined based on preliminary experiments and commonly used sample sizes in comparable publications within the field.<br>With in vitro data, sample size used for analysis was 3 - 6. For in vivo data generated using mice, 6-10 mice per group were used to evaluate the safety and efficacy of effects of CDK2 inhibition in AML. These sample sizes were sufficient to detect meaningful biological differences with good reproducibility.
Data exclusions	No data were excluded from the analyses.
Replication	To verify the reproducibility of our findings, experiments were performed using at least three biological replicates, unless clearly stated otherwise in the figure legends. All attempts at replication were successful.
Randomization	For in vitro experiments, control and treatment groups were derived from the same cell line, so no randomization could be performed.<br>For in vivo experiments, mice were randomly assigned to the described treatment groups
Blinding	For experiments involving human research participants, investigators were blinded to group allocation during data.<br>Investigators who performed animal experiments were not blinded because they needed to know how to treat mice with drug or placebo.But the Investigators were blinded during the data analysis.<br>Some in vitro cell culture experiments were not performed in a blinding manner because the same investigator performed the cell culture, treatment and processing. also, knowledge of these different conditions was required for sample preparation and analysis. But the Investigators were blinded during the data analysis. To ensure consistent experimental conditions, all control and experimental samples were processed in parallel.<br>All the data analyses were performed by an investigator blinded to the experimental conditions.

Reporting for specific materials, systems and methods

We require information from authors about some types of materials, experimental systems and methods used in many studies. Here, indicate whether each material, system or method listed is relevant to your study. If you are not sure if a list item applies to your research, read the appropriate section before selecting a response.

Materials & experimental systems

n/a	Involved in the study
☐	☒ Antibodies
☐	☒ Eukaryotic cell lines
☒	☐ Palaeontology and archaeology
☐	☒ Animals and other organisms
☐	☒ Human research participants
☒	☐ Clinical data
☒	☐ Dual use research of concern

Methods

n/a	Involved in the study
☒	☐ ChIP-seq
☐	☒ Flow cytometry
☒	☐ MRI-based neuroimaging

Antibodies

Antibodies used

The following antibodies were used (all commercially available):
From Proteintech:
1.beta-actin Monoclonal Antibody (66009-1-Ig,1:8000)
2.HSP90 Polyclonal Antibody(13171-1-AP,1:1000)
3.CDC37 Polyclonal Antibody(10218-1-AP,1:1000)
4.ATG7 Polyclonal Antibody(10088-2-AP,1:1000)
5.TRIM21 Polyclonal Antibody(12108-1-AP,1:1000)
6.RUVBL2 Polyclonal Antibody(10195-1-AP,1:1000)
7.DNAJA1 Polyclonal Antibody(11713-1-AP,1:1000)
8.Beclin 1 Polyclonal Antibody(11306-1-AP,1:1000)
9.GAPDH Monoclonal Antibody(60004-1-Ig,1:8000)
10.HSP70 Polyclonal Antibody(10995-1-AP,1:1000)
11.Cyclin A2 Polyclonal Antibody(18202-1-AP,1:1000)
12.GST Tag Monoclonal Antibody(66001-1-Ig.1:8000)
13.CDK1-Specific Polyclonal Antibody(19532-1-AP,1:1000)
From Cell Signaling Technology :
14.DDX5 (D15E10) XP® Rabbit mAb(#9877,1:1000)
15.Stathmin Antibody(#3352,1:1000)
16.Mcl-1 (D5V5L) Rabbit mAb(#39224,1:1000)
17.Anti-mouse IgG, HRP-linked Antibody(#7076,1:3000)
18.Anti-rabbit IgG, HRP-linked Antibody(#7074,1:3000)
19.HA-Tag (C29F4) Rabbit mAb(#3724,1:1000)
20.CDK2 (E8J9T) XP® Rabbit mAb(#18048,1:1000)
21. Cyclin E1 (D7T3U) Rabbit mAb(#20808,1:1000)
22.Nucleolin (D4C7O) Rabbit mAb(#14574,1:1000)
23.Rb (4H1) Mouse mAb(#9309,1:1000)
From Abcam:
24.Anti-Nucleolin (phospho T84) antibody (ab155977,1:1000)
25.Anti-Rb (phospho T821) antibody (ab4787,1:1000)
from Promega:
26. Anti-HaloTag antibody (G9281,1:1000)
From Sigma-Aldrich:
27.Monoclonal ANTI-FLAG® M2 antibody (F1804,1:5000)

Validation

Validation for commercially available antibodies can be found using the links below:
1.https://www.ptgcn.com/products/Pan-Actin-Antibody-66009-1-Ig.htm
2.https://www.ptgcn.com/products/HSP90-Antibody-13171-1-AP.htm
3.https://www.ptgcn.com/products/CDC37-Antibody-10218-1-AP.htm
4.https://www.ptgcn.com/products/ATG7-Antibody-10088-2-AP.htm
5.https://www.ptgcn.com/products/TRIM21-Antibody-12108-1-AP.htm
6.https://www.ptgcn.com/products/RUVBL2-Antibody-10195-1-AP.htm
7.https://www.ptgcn.com/products/DNAJA1-Antibody-11713-1-AP.htm
8.https://www.ptgcn.com/products/BECN1-Antibody-11306-1-AP.htm
9.https://www.ptgcn.com/products/GAPDH-Antibody-60004-1-Ig.htm
10.https://www.ptgcn.com/products/HSPA1A-Antibody-10995-1-AP.htm
11.https://www.ptgcn.com/products/CCNA2-Antibody-18202-1-AP.htm
12.https://www.ptgcn.com/products/gst-Antibody-66001-1-Ig.htm
13.https://www.ptgcn.com/products/CDC2-Specific-Antibody-19532-1-AP.htm
14.https://www.cellsignal.cn/products/primary-antibodies/ddx5-d15e10-xp-rabbit-mab/9877
15.https://www.cellsignal.cn/products/primary-antibodies/stathmin-antibody/3352
16.https://www.cellsignal.cn/products/primary-antibodies/mcl-1-d5v5l-rabbit-mab/39224
17.https://www.cellsignal.cn/products/secondary-antibodies/anti-mouse-igg-hrp-linked-antibody/7076
18.https://www.cellsignal.cn/products/secondary-antibodies/anti-rabbit-igg-hrp-linked-antibody/7074
19.https://www.cellsignal.cn/products/primary-antibodies/ha-tag-c29f4-rabbit-mab/3724
20.https://www.cellsignal.cn/products/primary-antibodies/cdk2-e8j9t-xp-rabbit-mab/18048
21.https://www.cellsignal.com/products/primary-antibodies/cyclin-e1-d7t3u-rabbit-mab/20808
22.https://www.cellsignal.cn/products/primary-antibodies/nucleolin-d4c7o-rabbit-mab/14574
23.https://www.cellsignal.cn/products/primary-antibodies/rb-4h1-mouse-mab/9309

24.https://www.abcam.cn/nucleolin-phospho-t84-antibody-epr8080-ab155977.html
25.https://www.abcam.cn/Rb-phospho-T821-antibody-ab4787.html
26.https://www.promega.com.cn/products/protein-detection/primary-and-secondary-antibodies/anti-halotag-pab/?catNum=G9281
27.https://www.sigmaaldrich.cn/CN/zh/product/sigma/f1804

Eukaryotic cell lines

Policy information about cell lines

Cell line source(s)	Source of all cell lines (THP1, A549, Hep3B, HEK293, HEK293T): ATCC.
Authentication	All cell lines have been authenticated by short tandem repeat analysis.
Mycoplasma contamination	All cell lines tested negative for mycoplasma.
Commonly misidentified lines (See ICLAC register)	No commonly misidentified cell lines were used.

Animals and other organisms

Policy information about studies involving animals; ARRIVE guidelines recommended for reporting animal research

Laboratory animals	C57BL/6 mice (6-8 weeks old female) were purchased from Charles River Laboratories.<br>NOD/SCID/IL-2γ-receptor null (NSG) mice (5-6 weeks old female) were purchased from Gempharmatech Corporation.
Wild animals	The study did not involve wild animals.
Field-collected samples	The study did not involve samples collected from the field
Ethics oversight	All animal experiments were performed with approval from the Institutional Animal Care and Use Committee of City of Hope Medical Center as well as Animal Ethical and Welfare Committee of ZCMU.

Note that full information on the approval of the study protocol must also be provided in the manuscript.

Human research participants

Policy information about studies involving human research participants

Population characteristics	Data involving human research participants pertain to Western blot of leukemia samples, and were de-identified prior to analysis
Recruitment	Not specific patients were recruited for this study. All clinical sample were collected in The Second Affiliated Hospital of Zhejiang University School of Medicine and were provided as unslected samples to ensure blinding approach.
Ethics oversight	All human tumor specimens were obtained under an Institutional Review Board approved protocol of the Second affiliated Hospital, School of Medicine, Zhejiang University.

Note that full information on the approval of the study protocol must also be provided in the manuscript.

Flow Cytometry

Plots

Confirm that:

- [x] The axis labels state the marker and fluorochrome used (e.g. CD4-FITC).
- [x] The axis scales are clearly visible. Include numbers along axes only for bottom left plot of group (a 'group' is an analysis of identical markers).
- [ ] All plots are contour plots with outliers or pseudocolor plots.
- [x] A numerical value for number of cells or percentage (with statistics) is provided.

Methodology

Sample preparation	Flow cytometry was used for cell cycle analysis using propidium iodide (PI) staining. After fixation of one million cells overnight at 4°C with 80% ethanol, cells were resuspended in PBS supplemented with 0.1% Triton X-100, 25 mg/ml PI, and 0.2 mg/ml RNase A, and incubated for 45 min at 37°C in the dark before analysis. Flow cytometry was used for cell apoptosis analysis using Annexin V-APC / 4',6-diamidino-2-phenylindole (DAPI) staining. Cells were resuspended in 100 µl Binding Buffer to a concentration of one million cells per ml. Then, 5 µl of Annexin V-APC and 1 µl DAPI solution were added to the sample and mixed gently. Samples were incubated for 15min at room temperature in the dark and further diluted with 400µl Binding Buffer prior to being assessed within 1h by flow cytometry.

Instrument	FACSCalibur flow cytometer (BD Biosciences)
Software	Data collection was done with BD CellQuest Pro sofware (BD Biosciences), and analysis was performed using FlowJo software.
Cell population abundance	No FACS sorting was performed
Gating strategy	Cell debris as well as non-singlets were excluded by a gate based on FSC-H vs SSC-H, FSC-W vs FSC-H, and SSC-W vs SSC-H.

☒ Tick this box to confirm that a figure exemplifying the gating strategy is provided in the Supplementary Information.
